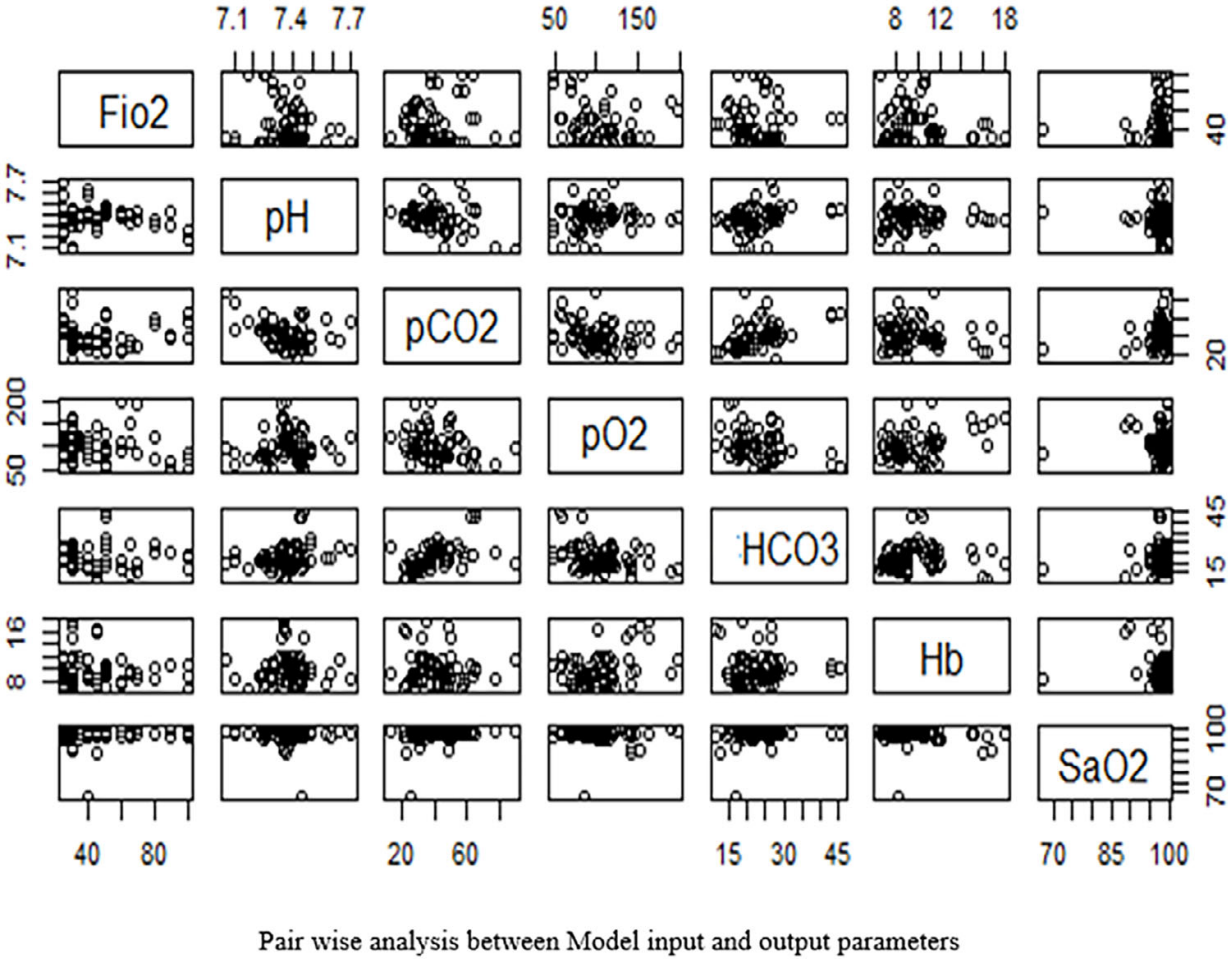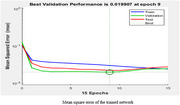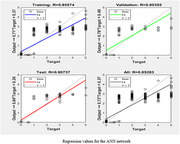# Artificial Neural Network Based Ventilator Support System for Automatic Respiratory Assistance to Alzheimer's Disease Patients

**DOI:** 10.1002/alz70858_096826

**Published:** 2025-12-24

**Authors:** Sita Radhakrishnan, Jagath Prasad Sreedhar G

**Affiliations:** ^1^ SAINTGITS College of Engineering, KOTTAYAM, KERALA, India; ^2^ University of Technology and Applied Sciences, Muscat, Muscat, Oman

## Abstract

**Background:**

A person suffering from dementia will eventually lose the ability to breathe if that part of the brain has been seriously affected. If left untreated, this will cause the victim to die. Continuous breathing assistance is necessary to regulate their breathing. In order to meet this requirement an automatic ventilator support system using artificial neural network (ANN) is proposed for getting the required blood oxygen saturation level (SaO_2_).

**Method:**

Samples from 325 people with acute respiratory issues, including dementia patients, were collected at ASTER Medcity in Kochi, India, with ethical permission. Parameter identification for model inputs and outputs is done by in cooperating real time patient data including periodical arterial blood gas analysis, continuous pulse oximetry readings and mechanical ventilator settings using statistical R programming. Artificial neural network model was developed using MATLAB programming to predict inspired oxygen (FiO_2_) and comparison also undertaken with physician's prediction.

**Result:**

After so many trial and error we got the mean square error of the trained model network as 0.019907 and R value as 0.852. To reduce over fitting hidden layer value must be taken less than twice the input layer size. New data set different from that used for modelling ANN was used for testing. Compared with the predictions of the physicians the artificial neural network model shows 86% accuracy

**Conclusion:**

Continuous oxygen saturation level monitoring and control is essential for Alzheimer's disease patient, since persistent respiratory diseases in these people can become a common reason for sudden death. For this a ANN model using MATLAB programming was developed to predict inspired oxygen amount which was given to the patient as life support. The suggested model output was found to be utilized as a suggestive method to assist clinicians because it displays an accuracy of over 80% when compared to the decisions made by physicians.